# Integration of Heterogeneous Materials for Wearable Sensors

**DOI:** 10.3390/polym10010060

**Published:** 2018-01-18

**Authors:** Yaser M. Haddara, Matiar M. R. Howlader

**Affiliations:** Electrical & Computer Engineering, McMaster University, 1280 Main Street West, Hamilton, ON L8S 4K1, Canada; howladm@mcmaster.ca

**Keywords:** heterogeneous integration, wearable sensors, flexible electronics, stretchable electronics, inkjet printing, surface-activated bonding

## Abstract

Wearable sensors are of interest for several application areas, most importantly for their potential to allow for the design of personal continuous health monitoring systems. For wearable sensors, flexibility is required and imperceptibility is desired. Wearable sensors must be robust to strain, motion, and environmental exposure. A number of different strategies have been utilized to achieve flexibility, imperceptibility, and robustness. All of these approaches require the integration of materials having a range of chemical, mechanical, and thermal properties. We have given a concise review of the range of materials that must be incorporated in wearable sensors regardless of the strategies adopted to achieve wearability. We first describe recent advances in the range of wearable sensing materials and their processing requirements and then discuss the potential routes to the integration of these heterogeneous materials.

## 1. Introduction

Wearable sensors have been a topic of interest for the last four decades, but the volume of research has dramatically increased in recent years with advances addressing the challenges of materials, devices, energy, and information storage and processing.

**Figure 1:**
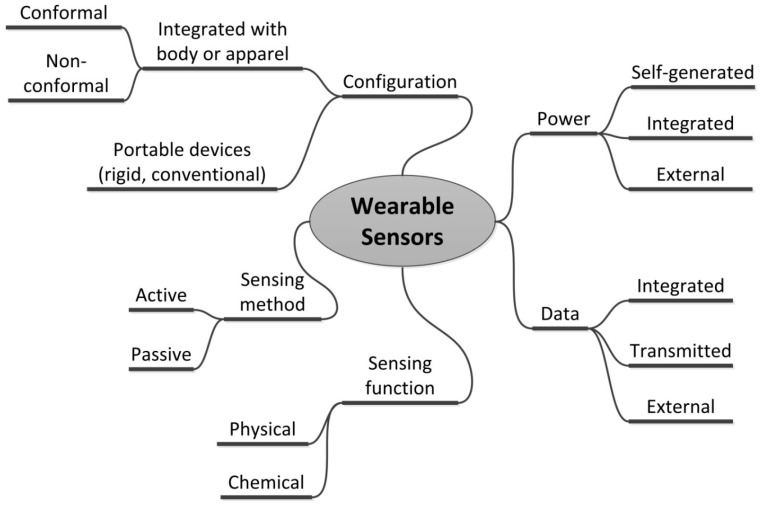
A conceptual categorization of wearable sensors. The configuration of the sensor on the body and the strategies adopted for power and data are issues specifically related to wearability. Sensing function and method are issues pertinent to all sensors.

As we show in Figure 1, there are several considerations that may be used to categorize wearable sensors. Most pertinent in terms of wearability is the configuration of the sensor on the body. The label “wearable” has been applied to electronics in general and sensors in particular in two distinct ways. The first refers to devices that are already commercially ubiquitous, such as smart watches and pedometers. These rely primarily on conventional semiconductor technologies and are the result of the aggressive scaling that has characterized the development of the semiconductor industry since its inception. While certainly wearable, these tend to be rigid devices that may be more appropriate to think of as portable. The second way in which the term wearable is used refers to sensors that are more integrated with a person’s body or apparel and are, preferably, less obtrusive on the person than a portable device. There has been a wide range of applications suggested for such sensors but the most active research today focuses on health and environmental applications. Sensors thus integrated with the skin or apparel may be further subdivided by whether a sensor is intended to be in intimate contact with the body, in which case the device must conform to the movement of the wearer, or whether it is non-conformal. For non-conformal configurations, most applications still require the device to be integrated with apparel, so flexibility and imperceptibility are still required.

Two other considerations relevant to wearability are power and data. Wearable sensors may generate their own power using some method of energy harvesting or generation, may have an integrated power source (i.e., some form of battery), or may be dependent on external connection for a power supply at the time of measurement. In terms of data, the issues relate to storage, processing, and analysis of data. Any range of these functions may be integrated with the device. Alternatively, data may be transmitted to a smart device for analysis and display or connected to an external system for data acquisition and analysis at specific measurement times. Finally, wearable sensors may be categorized as any other sensor in terms of their sensing function (physical or chemical) and type of measurement (active or passive).

As mentioned above, for wearability in the sense of integration with body or apparel, flexibility is required and imperceptibility is desired. It is this aspect of wearability that is of primary interest in this paper. We will consider the range of materials needed to design and fabricate wearable, flexible sensors. We will not limit our focus to any one category or focus on the distinctions around configuration, conformality, power and data requirements, or type or method of sensing.

There are multiple challenges that must be addressed before truly wearable sensors can become practical and ubiquitous, not least of which are the challenges of power generation and data storage, communication, and analysis. Several critical and comprehensive reviews have appeared recently that address the topic of wearable sensors from multiple angles, including sensing mechanisms, strategies for stretchability, current challenges, and system integration [1,2,3,4,5,6,7,8]. There has been a range of strategies proposed to achieve wearability together with reliability and robustness, and there have been particularly significant innovations in materials, processes, structures, and integration as well as innovative use of a range of material properties to make measurements relevant to personal activity and healthcare monitoring. 

In this paper, we do not focus on the types of sensors, the approaches to stretchability and flexibility, the mechanisms of operation of the sensors, the challenges still to be addressed, or material selection and sensor design. Each of these aspects has been comprehensively reviewed in one or more of the studies cited above. However, it is clear that regardless of the particular sensor, specific physical and/or chemical mechanism, and the selected approach to achieve flexibility and/or stretchability, wearable sensors are going to require the integration of heterogeneous materials: multiple materials with a wide range of thermal, mechanical, and electrical properties. It is this requirement for integration that is the focus of this paper. In Section 2, we give a concise survey of the range of materials required. The intent is not to be comprehensive in listing all the specific candidate materials for sensor applications or to address issues of material selection or the tailoring of material properties. Rather, the intent is to survey the full range of the kinds of materials that are used and the applications for which they are needed. We have included sufficient examples to illustrate both the range of properties as well as the range of designs for flexible, stretchable sensors. In Section 3, we then address the methods being explored to achieve low-cost integration of heterogeneous materials to produce durable, flexible, and stretchable wearable sensors.

## 2. Materials for Wearable Sensors

For the most part, wearable sensors rely on the same sensing mechanisms implemented in conventional or portable measurement equipment. An examination of the literature shows a wide range of sensing mechanisms that have been attempted with materials that have the requisite properties, including the use of pyroelectric materials or thermocouples to measure temperature, piezoelectric materials to measure pressure or strain, electrolytic cells to measure chemical concentration, cantilevers to measure movement, as well a range of other properties, structures, and mechanisms [2]. The challenge in each case is to integrate the required heterogeneous materials onto a single platform such that the overall structure is flexible and robust. The materials used include metals, conventional semiconductors such as silicon, flexible polymers, textiles, and 2D materials. We will consider examples of each of these, together with the strategies used to achieve flexibility, reliability, and imperceptibility.

### 2.1. Metals

Metals are required in any electronic system, at least to make contacts. Additionally, metals have been used for specific sensing functions, the most important being measurements of temperature [1,9,10,11] and of chemical concentrations of specific analytes using voltammetric methods. Voltammetry has also been used with palladium/palladium oxide electrodes (Pd/PdO) to detect pH [12].

For chemical analysis, much of the research focuses on different voltammetry configurations. Voltammetry relies on three electrode devices: a working electrode (WE), a reference electrode (RE), and a counter electrode (CE). For a given analyte, the choice of electrode, the geometry of the device, the processing to obtain a clean sensing surface and protect it from contamination and degradation, and the voltammetry configuration, are studied and optimized.

Square wave anodic stripping voltammetry (SWASV) has been used to detect the concentration of heavy metals in body fluids. The choice of working electrode is critical to selectivity, sensitivity, and stability. Mercury (Hg) has been widely used but is unsuitable for wearable sensors due to its toxicity and volatility. Gold (Au) working electrodes have been shown to have an excellent response for the detection of lead (Pb), copper (Cu), and mercury (Hg) in human sweat while bismuth (Bi) is ideally suited for the detection of Pb, cadmium (Cd), and zinc (Zn).

Gao et al. [9] fabricated a multiplexed microsensor array using Au and Bi working electrodes, a silver (Ag) reference electrode, an Au counter electrode, and chromium/gold (Cr/Au) microlines for temperature sensing. The Au/Bi working electrodes provide a response that simultaneously detects Pb, Hg, Cu, Zn, and Cd concentrations in human sweat. They demonstrated the independence of these signals to the presence of multiple heavy metals and calibrated the measurement to the measured skin temperature from the Cr/Au sensor. The array can be calibrated using off-body measurements and then worn in a wristband for real-time, on-body monitoring. Yeo et al. [11] used Ti/Pt for temperature measurement. Matsuzaki and Tabayashi [13] used thin films of gallium indium tin (GaInSn) electrodes sandwiched between layers of polydimethylsiloxane (PDMS) to measure local and global strain to sense the folding of multiple finger joints.

While silver/silver chloride (Ag/AgCl) electrodes have been extensively used for reference and counter electrodes in voltammetry setups, there has been recent effort to explore the use of Cu-based electrodes for all three electrodes in a voltammetric sensor [14]. If problems of durability and adhesion can be overcome, Cu electrodes become attractive due to low cost and good conductivity. Cu electrodes have been shown to be effective as sensors for Pb and may be suitable for the detection of other heavy metals. Redhwan et al. have recently demonstrated a Cu-based sensor by bonding Cu to a liquid crystal polymer (LCP) substrate using surface-activated bonding (SAB) [15]. Howlader et al. [16] have also given a review of different deposition methods for Cu electrodes in sensing applications.

Thin films of other metals deposited on flexible substrates have also been explored by a variety of methods. Qin et al. gave a review of different materials used, whether for the sensing function or as flexible substrates (See [17] and references therein). Much of the current research in this direction is focused on optimizing and characterizing the sensing behavior, developing methods for the integration of different materials, and assessing the robustness and stability of the sensors under various physical and environmental stimuli.

In addition to chemical and temperature sensors, thin metal films have also been used in a wide range of other applications. Silver nanowires (AgNW) have been used for motion sensing [18] while Cr/Au filaments have been used to measure electrophysiological signals [11]. 

In these and other studies, flexibility is obtained by using sufficiently thin films as well as the design of the structure to achieve flexibility. Design approaches include the use of open filamentary serpentine structures, wavy out-of-plane structures, or sandwiching the films between layers of elastomeric material so that the films are in the neutral mechanical plane (NMP). The importance of constructing the NMP has been described in [8] together with a systematic discussion of material selection and optimized configurations.

### 2.2. Conventional Semiconductors

Sufficiently thin layers of inorganic semiconductors such as silicon (Si) can also exhibit sufficient flexibility for wearable electronics. Kim et al. [19] reported on the design and fabrication of stretchable and foldable Si integrated circuits (ICs). The circuits are designed on a sacrificial poly(methylacrylate) (PMMA) substrate. A layer of polyimide (PI) is then deposited and nanoribbons of doped Si are transfer printed on the PI using an elastomeric stamp. Conventional fabrication steps are then used to pattern Si, deposit oxide, and form interconnects. An array of holes approximately 50 um in diameter and spaced 800 um apart are etched in the material using reactive ion etching. The holes are made into non-functional regions of the wafer, penetrate to the PMMA layer, and are used to deliver solvent to the PMMA layer to release the PI flexible layer with electronics formed. The circuits thus released can be used as a foldable, stretchable sheet, or attached to elastomeric substrates, such as polydimethylsiloxane (PDMS), as a wavy stretchable and compressible product.

Yeo et al. [11] used Si nanomembrane strain gauges in their multisensor system. The nanomembranes were first fabricated on a silicon-on-insulator (SOI) wafer. They were then transferred to a bulk Si wafer and conventional semiconductor fabrication techniques were used to fabricate additional structures. Si nanomembranes are not flexible components. However, the small dimensions and integration with the rest of the electronics in an open mesh structure allowed the incorporation of brittle Si into a system that was flexible and robust. 

### 2.3. Polymers

The most important use of polymers in the fabrication of wearable sensors is as flexible substrates for the overall system. Polydimethylsiloxane (PDMS) and polyimide (PI) are probably the most frequently used [11,17,20,21,22,23]. The review by Qin et al. [17] surveys other polymers that have been suggested as substrates and gives a comparison of their properties.

The second research direction for polymers in sensing applications is the use of polymer electronics, and in particular polymer transistors, including polymer dielectric layers and conductors [24,25,26,27,28,29]. A number of active organic materials have been used to successfully fabricate polymer transistors, including poly(3,4-ethylenedioxythiophene) doped with polystyrenesulfonate (PEDOT:PSS) [25] and poly(3-alkyl thiophenes) (P3AT) [24]. These devices are attractive because of the potential for large area fabrication on flexible substrates and because they can integrate the sensing functions with the electronics through the impact that analytes can have on the channel properties, either of the organic material as-is or through the addition of functional groups (for a comprehensive review see [28]). For example, PEDOT:PSS has been used as the active layer in an organic electrochemical transistor (OECT) for detection of glucose in blood serum [30]. Conducting polymers have also been used as electrodes [31,32,33].

However, there have been a number of applications that exploit properties of flexible organic polymers for the sensing function itself. One particularly promising material is poly(vinylidenefluoride) (PVDF) and its copolymers. In particular, poly(vinylidenefluoride-*co*-trifluoroethylene) [P(VDF-TrFe)] is a material that has shown excellent piezoelectric behavior for pressure and strain sensing as well as being suitable for temperature sensing [34,35,36,37,38]. Persano et al. [34] demonstrated a method for electrospinning fiber arrays with high crystallinity and fiber alignment, resulting in a very strong piezoelectric response (on the order of mV/Pa). The electrospinning method involves spraying the polymer, dissolved in a slow evaporating solvent from a 200 um nozzle, towards a spinning collector with an electric field between the nozzle and the collector. In addition to the nozzle diameter, process parameters to optimize are the flow rate of fluid into the nozzle, the distance of the nozzle to the collector, the boiling point of the solvent, the applied electric field, and the spin speed of the collector. With an optimal process, fibers overlap during spinning and form joints at the mesoscale that promote the alignment of fibers and crystallinity of the overall array. These ribbons may be woven into bands or other wearable textiles which then act as the pressure sensors. These are responsive to static or dynamic pressure as well as to measurements of strain due to bending or twisting. The piezoelectric response also allows human motion sensing based on PVDF.

The ferroelectric and pyroelectric properties of PVDF and P(VDF-TrFe) also allow for their use as touch [39,40] and temperature sensors [35]. Xue et al. [35] demonstrated the use of PVDF for energy harvesting and as a self-powered temperature sensor for human breathing. By coating with a Pd film, it can also be used as a hydrogen sensor [41].

### 2.4. Smart Textiles

Smart textiles push the idea of wearable sensors to potentially transform actual apparel into a sensing platform. While much research still remains to make the concept practical, there has been significant progress in developing fibers with sensing functions that may be woven into clothing. We have already discussed the work of Persano et al. [34] in the previous section. Wang et al. [42] fabricated fiber OECT devices using polypyrrole/reduced graphene composites on PA6 fiber filaments. Soltanian et al. [43] fabricated piezoelectric nanofibrous strain sensors and Badawy et al. [44] reported on piezoelectric zinc oxide woven fabric sensors. Other groups have also reported on weaving/knitting wearable sensors based on different materials/yarns [45,46,47].

The fabrication processes for these textiles are multistep processes involving the coating of a nanofiber core material with successive layers of material and, where needed, in situ polymerization, in addition to cooling, heating, and drying steps depending on the material used. Once the composite fiber has been fabricated, it may be woven or knitted as desired.

Applications that have been demonstrated span a wide range including glucose measurement, hydration monitoring, respiratory rate monitoring, and motion and tactile detection. The sensors in [43,44] are proposed as part of a system that could, for example, use the measurement to control applied pressure to muscles by a medical brace. Servati et al. have given a comprehensive review [48].

### 2.5. Two-Dimensional (2D) Materials

Two-dimensional (2D) materials possess unique mechanical, electrical, optical, and chemical properties [49,50]. The electronic structure of each material can be tailored, resulting in a semimetal, semiconductor, or insulator. Two-dimensional materials can broadly be categorized into intrinsic materials such as graphene, functionalized materials such as graphene oxide and graphene-metal composites, transition metal dichalcogenide-based materials such as molybdenum disulfide and tungsten disulfide, and III–VI semiconducting materials such as gallium sulfide, black phosphorus, and hexagonal boron nitride [49,50]. Due to their atomic-thin layered structure, large surface-to-volume ratio, large adsorption capacity of gas molecules and strong surface chemical activities, 2D materials have been demonstrated for gas sensing in health, environment, and security applications. Specifically, these materials have considerable demand as wearable gas sensors due to real-time detection capabilities of hazardous gases such as ammonia (NH_3_), explosives such as 2,4-dinitrotoluene gas (DNT), and chemical weapons such as dimethyl methylphosphonate (DMPP) gas, allowing the wearer to identify potential hazards and terrorism threats in the local environment. While 2D materials are critical for wearable gas sensors, a relatively high operating temperature generally limits their applications.

The room temperature sensor operability of graphene, with its high carrier mobility of 2 × 10^5^ cm^2^·v^−1^·s^−1^ [51], carrier density of 10^12^ cm^−2^ [52] and fracture strength of 130 GPa [53], and low resistivity and noise, makes it a next generation high-performance wearable gas sensing material. Here, we briefly focus on graphene-based wearable gas sensors operated at room temperature. The structures for these sensors are mainly based on chemiresistor, field effect transistor, and surface acoustic wave configurations for sensing different hazardous gases. We provide some examples of the sensors for gases including nitrogen dioxide (NO_2_), NH_3_, DNT, DMPP, hydrogen (H_2_), hydrogen sulfide (H_2_S), carbon dioxide (CO_2_), and humidity in wearable sensing technology. Unlike typical metal oxides (e.g., zinc oxide), the sensing mechanism of graphene-based 2D materials is based on a charge transfer mechanism.

High-quality intrinsic graphene can be prepared for gas sensing using mechanical exfoliation and chemical vapor deposition (CVD) techniques. The preceding one has limited scalability due to film exfoliation of below 1000 um^2^. In contrast, the latter one offers large-area synthesis of graphene films. In CVD, graphene is prepared on metal substrates such as Cu at temperatures below 1000 °C and transferred onto flexible substrates such as PI [54]. The CVD-grown pristine graphene films using in situ cleaning with ultraviolet light were exploited to fabricate the chemiresistor-type high sensitivity sensors [55]. Recently, Kim et al. [54] demonstrated a self-activated transparent CVD-based all-graphene gas sensor on PI substrate for NO_2_ and humidity. These sensors endured humidity and mechanical bending. The sensors showed reversible responses to NO_2_ without external heating under high humid conditions and bending strain. In another study [56], CVD-grown graphene was directly transferred onto paper. The sensors were robust to minor strain. Therefore, these sensors can be used for wearable gas sensing applications.

Functionalized graphene can usually be synthesized from purified graphite powder by heavily oxidizing it using modified Hummers method [57] and then chemically reducing it, resulting in reduced graphene oxide (rGO) with partially restored conductivity [58]. The resulting rGO films have defect sites with functional groups acting as adsorption sites. Currently, major chemical-reducing agents being used include hydrazine, ascorbic acid, *p*-phenylenediamine (PPD), and sodium borohydride (NaBH_4_). The reducing agents have high, mild, weak, and non-toxic behavior, respectively. Hydrazine-based rGO sensors showed sensitivity of 52 ppb for 2,4-dinitrotoluene gas (DNT) [59]. It is worth noting that DNT is a volatile component of trinitroluene explosives. Ascorbic acid based rGO sensors on PET substrate fabricated using inkjet printing techniques showed reversible and selective gas detections of NO_2_ and Cl_2_ in the range of 100 ppm to 500 ppb at room temperature [60]. In order to investigate hazardous gases such as dimethyl methylphosphonate (DMPP) being used in chemical weapons, PPD-based rGO sensors were fabricated [61]. These sensors showed three times higher sensitivity than that of hydrazine-based rGO sensors for sensing 30 ppm of DMPP gas. The NaBH_4_-based rGO exhibited exceptional sensitivity of 5.5% at 200 ppm and 23% at 2800 ppm of NH_3_ gas without heat treatment, having quick resistance recovery [62]. Also, multilayer graphene was developed onto a filter paper to fabricate a cost-effective and highly sensitive sensor for the detection of NH_3_ gas [63]. The sensor detected NH_3_ concentrations as low as 430 ppb. The sensor exhibited a response of ~3% at 400 ppm and ~13% at 1200 ppm of NH_3_ gas. The variation in sensitivity was verified for both flat and bent positions. A chemically fluorinated GO showed fast response/recovery behavior and high sensitivity of detecting 2 ppm NH_3_ and 100 ppm NH_3_ with ~3.8% response in 30 s at room temperature [64]. Lately, a study on the functionalization of graphene surface showed that chemical stability and interaction can be controlled by choosing the type of treatment. While fluorinated graphene and oxygenated graphene-based sensors exhibited similar sensitivity to NH_3_ and NO_2_ gases, the preceding one provided better recovery due to its higher chemical stability and weaker interactions with the adsorbed molecules [65].

Functionalized grapheme-nanomaterial composites have been prepared by incorporating functional nanomaterials, including metals, metal oxides, carbon nanotubes (CNTs), and polymers, into graphene networks forming hybrid structures. Graphene-nanometal composites offer higher sensitivity and selectivity due to specific catalytic behavior of the noble metals-like palladium (Pd) and silver (Ag) nanomaterials for gas sensing. For example, rGO films onto Ag nanoparticles (rGO/AgNPs) and Ag nanowires (rGO/AgNWs) exhibited higher sensitivity for NH_3_ gas compared with rGO/AgNPs and rGO sensors at room temperature [66]. These sensors also showed excellent responses and recovery characteristics. Recently, CVD grown graphene was doped with AgNPs and charged impurities by dipping in AgNO_3_ and Fe(NO_3_)_3_ solution to create a highly sensitive chemiresistive sensor for H_2_S. In another study [67], Ag NWs coated by an rGO layer on PET substrate were immersed in a blended suspension of aniline and rGO to deposit rGO/PANI composite through polymerization. Then, these rGO/PANI films were used to assemble supercapacitors. They exhibited enhanced capacitance and superior transparency at visible light with high stability and flexibility. Therefore, these can be used for integrated wearable sensing systems.

For Pd–rGO composite sensing materials, an electrodeposited Pd on diaminonaphthalene treated CVD graphene process was developed onto poly(ethylene-2,6-naphthalene dicarboxylate) (PEN) substrate [68]. This results in flower-like Pd nanoclusters on graphene electrodes, which was ultrasensitive to H_2_ gas sensing. These Pd–rGO sensors detected H_2_ gas as low as 0.1 ppm at room temperature. The sensitivity and response time of these sensors increased with increasing Pd nanoclusters [68]. For metal oxide–graphene composite, cuprous oxide (Cu_2_O) NWs–rGO sheets were prepared under hydrothermal conditions for NO_2_ gas sensing [69]. The sensitivity and limit of detection of this sensor for NO_2_ gas were 1.2 ppm and 64 ppb respectively at room temperature. It also exhibited a higher response than that of an individual Cu_2_O based sensor [69]. Finally, a graphene and PEDOT:PSS conducting polymer solution was used as ink to fabricate the sensor onto a flexible substrate to detect NH_3_ gas [70]. Analytical techniques confirmed a few-layer graphene in PEDOT:PSS matrix and π–π interactions occurring between graphene and PEDOT:PSS. The sensor showed high selectivity to NH_3_ in 25–1000 ppm at room temperature. This can be attributed to the increased specific surface area by graphene and increased π electron interactions between the sensing film and molecular NH_3_ [70].

### 2.6. Summary

We have given a brief survey of the range of materials being explored for wearable sensor applications. This Section is not intended to be a comprehensive review of all materials used in wearable sensor applications, the mechanisms of operation of the different sensors, or the relative performance of different sensor designs. Instead, what we have sought to emphasize is that regardless of sensor design, sensor functionality, and fabrication techniques, any wearable sensor must incorporate multiple materials that are widely heterogeneous in their thermal, mechanical, and electrical properties. One of the key challenges in fabricating wearable sensors is the integration of such materials in reliable, durable structures. Table 1 provides a summary of the range of materials surveyed in this Section.

## 3. Integration Strategies

The key challenge of working with these different materials and the requirements to create wearable, flexible, reliable sensor systems is the integration of heterogeneous materials and the fabrication of the innovative structures that have been discussed so far.

Using conventional process technology with heterogeneous materials covering a wide range of physical properties and the requirements of the innovative structures required for flexibility, poses a number of challenges. High temperature processes are not suited to structures that include organic materials, whether as flexible substrates or for electronics or sensing functionality. In addition, while patterning resolution is not an issue for most of these systems, alignment of different layers is critical in many of the proposed structures. A number of studies have focused on the manufacturability of flexible wearable sensors using conventional semiconductor process technology, including the fabrication of all organic electronic circuits [28] and the patterning of microfluidic channels in polymers such as PDMS [20]. Gao et al. [9] used conventional, complex fabrication processes for their sensor array. Fabrication is on a flexible polyethylene terephthalate (PET) substrate. The electrodes are deposited by e-beam evaporation and patterned by photolithography and liftoff. Parylene is used as an insulation layer, naphion is deposited on top of the structure to prevent biofouling and a polydimethylsiloxane (PDMS) well is used for sweat collection. For metal films on flexible substrates, a range of deposition and patterning techniques have been explored. Bismuth electrodes have been demonstrated for heavy metal detection by electrodeposition on flexible polyimide after surface modification by reduced graphene oxide and carbon nanotube composites [71] and by electroplating onto inkjet-printed electrodes on polyester [72]. Gold electrodes for a variety of applications have been deposited on flexible substrates by e-beam evaporation [73,74], inkjet printing [75,76], and electrodeposition [77]. 

**Figure 2:**
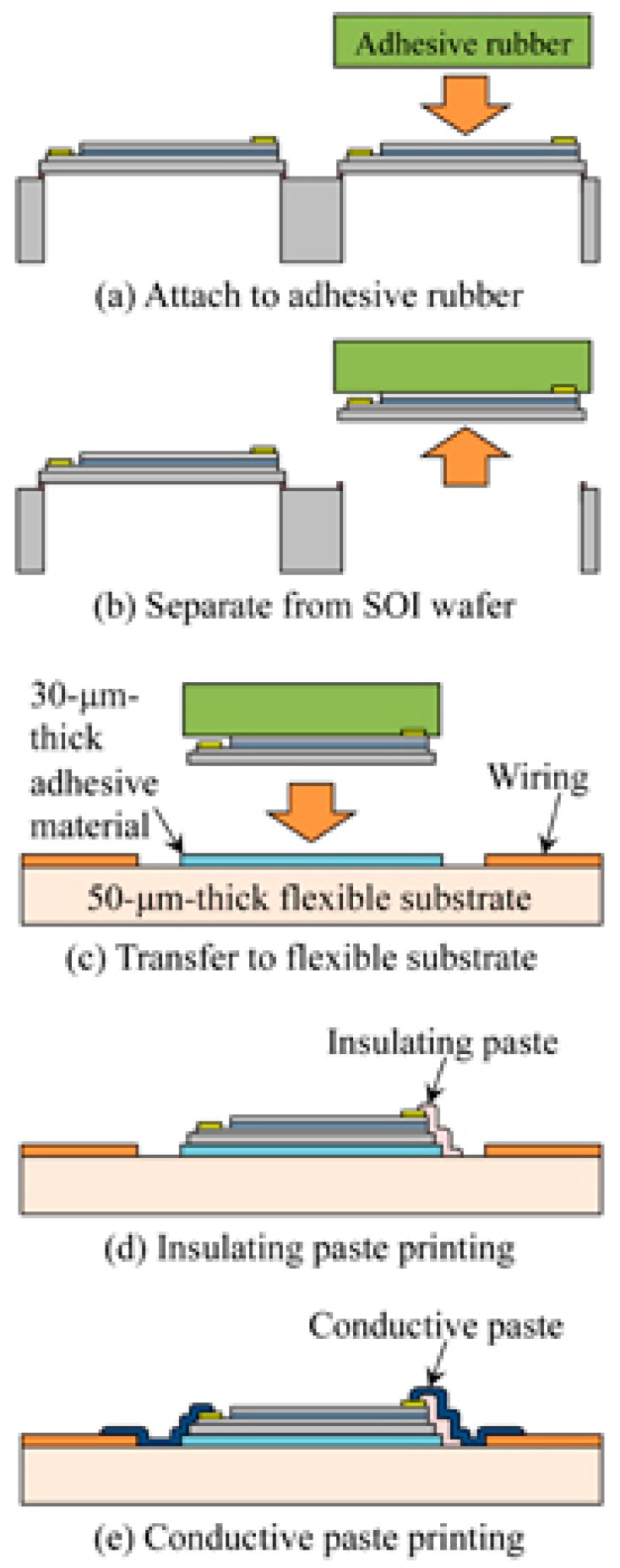
A schematic of the transfer printing process demonstrating the multi-step approach of patterning the device on a handle wafer followed by the release and transfer of the device to a flexible substrate using an elastomeric stamp or adhesive. From [79] (Copyright 2015 The Japan Society of Applied Physics).

Some groups have combined conventional fabrication steps with multiple transfer and bonding steps [78]. An example is the epidermal electronic systems (EES) approach demonstrated by Yeo et al. [11]. They demonstrated a multifunctional measurement system printed directly on the skin, comprising flexible components (polyimide and liquid bandage both used for encapsulation), flexible thin metallic films (Cr/Au for electrical measurements and Ti/Pt for temperature measurement), and Si nanomembrane strain gauges. The overall structure is an open mesh filamentary serpentine structure that enhances the deformability of the thin metal filaments as well as allowing the integration of brittle Si components. Another key consideration in the design was to use polyimide (PI) layers to encapsulate the overall system. With the exception of Au electrodes placed in direct contact with the skin for electrophysiological (EP) measurements, the electronics were encapsulated by two layers of 0.3 um thick PI placing the electronics at the neutral mechanical plane during deformation. The fabrication process involved three distinct processes. The first was the fabrication of the Si NM strain gauge on an SOI wafer. The second was the transfer of the strain gauge to a handle Si wafer followed by the fabrication of the remaining electronic system using conventional methods of lithography, sputtering, and etching. Finally, the device was transfer printed onto the skin via either an elastomeric stamp or a polyvinyl alcohol (PVA) water soluble sheet. Yamashita et al. [79] used transfer printing to place an ultrathin strain sensor on a flexible substrate. These different studies demonstrate the essential features of the transfer printing process; it is a multi-step process that involves the patterning of the active device or system on a handle substrate followed by the release and transfer of the device using an appropriately selected stamp or adhesive layer. Figure 2 (from [79]) shows a schematic of the process.

These approaches all suffer from significant complexity that affects reproducibility as well as cost. Two promising techniques for the direct integration of heterogeneous materials for fabrication of wearable sensors are inkjet printing and surface-activated bonding. This section of the paper provides a summary of the potential of these methods as well as the challenges to further development.

### 3.1. Inkjet Printing

Inkjet printing has also been explored as a means of integrating the required materials and fabricating structures for integrated sensors [80,81,82,83,84]. The primary challenges for inkjet printing relate to (1) the design of the ink to obtain uniform droplets and lines without waviness or buckling; (2) the use of inks that are biocompatible and low-temperature processable; and (3) achieving good adhesion to different substrates, particularly flexible substrates suited to wearable applications. 

For ink design, there are two broad categories of requirement: requirements related to achieving high printing performance and requirements specifically related to wearability. For printing performance, the primary considerations are ink composition (particle-based or other), viscosity, and surface tension. Particle-based inks have to be designed to fit the requirements of the particular printing head used to avoid clogging and pattern non-uniformities. The main factors affecting clogging are particle size, solvent evaporation, and ink dispersion. For droplet formation and line uniformity, a balance is required between ink viscosity and surface tension. The Ohnesorge number (*Oh*) is a measure of the ratio of viscosity to surface tension. Specifically, $Oh=\frac{\eta }{\gamma \rho \alpha }$ where η is the viscosity, γ is the surface tension, ρ is the density, and α is the droplet parameter. The *Z* parameter is given by $Z=\frac{1}{Oh}$ and is used to characterize printability. Typically, a value between 1 and 10 indicates good printability. At low values of *Z*, the high viscosity of the ink hinders drop ejection. At high values of *Z*, the primary droplet is accompanied by a number of satellite droplets, which is detrimental to line uniformity [85].

Inks for conductors and semiconductors must undergo post processing to improve conductivity. Thermal sintering, the predominant post processing method in other applications, is not suitable for wearable sensor applications where the materials involved have a wide range of thermal properties. In particular, flexible substrates are typically made of polymers that have low glass transition temperatures. Hence, alternate sintering methods have been explored, including chemical, electrical, photonic, and microwave sintering [81]. 

An attractive line of research has been the design of inks that do not require post annealing. Grouchko et al. [86] added NaCl to Ag nanoparticles capped with a polymer stabilizer (required to prevent ink dispersion and clogging). The NaCl acts as a destabilizing agent that removes the polymer during drying in a self-sintering process. Shin et al. [87] designed a Ag-salt based ink that is low temperature processable and does not require a capping agent, hence eliminating the need for a sintering step. The issues for conductive inks have been thoroughly discussed in [84]. Issues for dielectric and semiconductor inks have been covered in [80]. 

An example of conductive ink design for wearable applications uses a palladium based ink that is discussed in [82]. The resulting film morphology has been discussed in [83]. The authors observed that while adhesion of the resulting film to a glass substrate degraded for higher film thickness, the adhesion to a flexible plastic substrate was excellent for all film thicknesses. However, this effect was most likely the result of the particular ink chemistry used. This indicates part of the complexity in designing inks to achieve good adhesion to the particular substrate that will be used.

Issues for dielectric and semiconductor inks have been covered in [80]. Inorganic semiconductor inks pose the same challenges as for metallic inks (e.g., [88,89]). Organic inks exacerbate the challenge of thermal processing. Considerable work has been done in developing inks that can be processed at low temperatures without the requirement of high temperature annealing (e.g., [90,91,92]). Vescio et al. [93] have demonstrated the versatility of inkjet printing for integrating different materials. They demonstrated a fully printed metal–dielectric–metal structure using HfO_2_ as the dielectric and were able to achieve high uniformity, homogeneity, and structural integrity.

**Figure 3:**
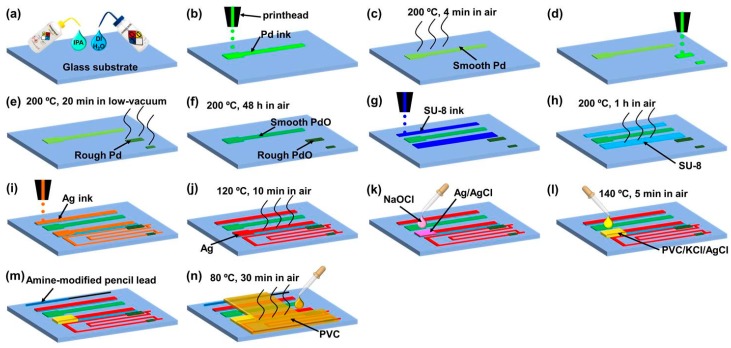
Inkjet printing process for an integrated sensor for pH, chlorine, and temperature [12].

Figure 3 shows a schematic of an inkjet printing process used to fabricate an integrated system for monitoring pH, free chlorine concentration, and temperature, to assess water quality [12]. The schematic shows the printing of multiple layers and the different thermal steps required. For a flexible substrate, alternate sintering methods would have to be used to allow processing at lower temperatures, as discussed above.

Beyond the design of the ink, there are significant issues for integration of the range of materials required for wearable sensors. Specifically, printing needs to be at a resolution sufficient to define the required circuitry and must allow the fabrication of flexible and stretchable structures. Resolution has been addressed either by modifying the wettability of the substrate (e.g., using a fluorine treated polyimide substrate [94] to ensure the definition of fine lines) or by modulating the chemical composition of the ink to ensure that the solute is restricted to the center of the drop and that drying does not cause further diffusion of the ink on the substrate (see e.g., [95]). In addition, it has been demonstrated that deposition parameters such as droplet spacing have an impact on line width, with an upper limit imposed by the requirement for continuous coalescence of a printed line and a lower bound imposed by bulging [96].

Flexibility relies on the use of flexible substrates. In the case of plastic substrates, we have already described some of the challenges. Textile substrates suffer the same challenges in addition to some specific challenges with ink adhesion and line continuity. The main approaches to addressing these issues have been either the coating of the textile with a layer suited to printing [97] or the formulation of specialized inks [98].

For stretchability, the mechanisms outlined in Section 2 have all been used with inkjet printing. The use of the neutral mechanical plane (NMP) is achieved by placing the active components between layers of flexible materials. Open filamentary structures and out of plane printing have also been utilized.

### 3.2. Surface-Activated Bonding

**Figure 4:**
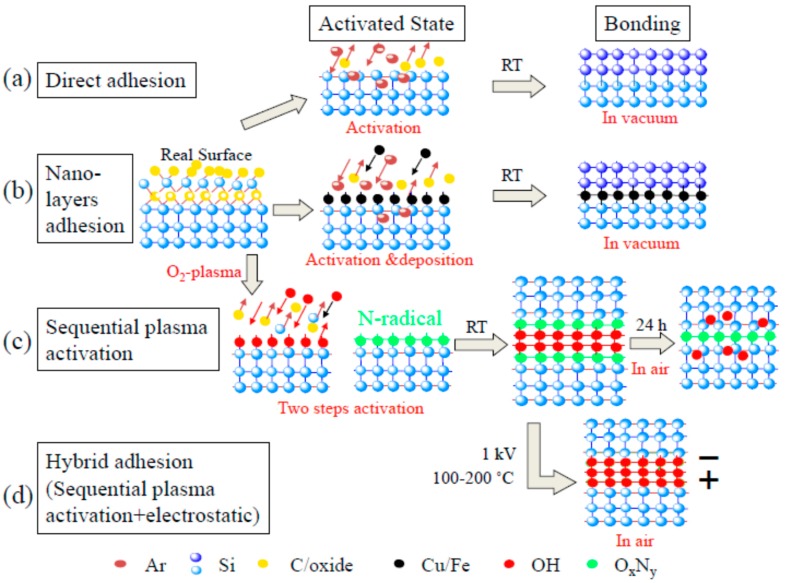
Surface-activated bonding of heterogeneous materials [103].

Finally, surface-activated bonding (SAB) is a promising low-cost technology that requires neither high temperature nor high pressure. Combined with simple etching or liftoff techniques for patterning layers, it can be used for simple sensor structures incorporating heterogeneous materials. It has been demonstrated for systems involving metals on flexible substrates [99,100] as well as for the integration of different conventional semiconductor materials [101,102]. As Figure 4 shows, in SAB, surfaces of different materials form a strong bond when they are brought into contact after activation by plasma bombardment [103].

Figure 4 shows different surface-activated bonding (SAB) approaches. SAB may be done in vacuum or in air. SAB in vacuum can either be (a) direct bonding in ultrahigh vacuum (UHV); or (b) nanolayers adhesion in UHV and HV. SAB in air includes (c) sequential plasma activation; and (d) hybrid adhesion (sequential plasma activation + electrostatic). These classifications are based on achieving optimal adhesion, controlled by surface and bulk properties of the bonding materials.

In the first approach, both surface-cleaning of mating materials using an argon fast atom beam (Ar-FAB) and bonding is accomplished in UHV. This technique mainly offers bonding of hard materials, resulting in covalent bonding [104,105,106,107,108]. In the second approach, the surfaces are simultaneously cleaned and nanoadhesion layers are deposited using a low energy Ar ion source and then brought into contact in UHV [109,110,111,112]. Different nanoadhesion layers such as Fe, Si, and Al have been investigated for different flexible polymers. These thin layers offer enhanced adhesion, specifically for the bonding of ionic materials [108,109,110,111]. Argon radio-frequency (Ar RF) plasma can also be used to clean the flexible surfaces and bond them in HV [99,100,113]. In the third approach, the surfaces are sequentially cleaned using oxygen reactive ion etching plasma followed by nitrogen microwave plasma in low vacuum then brought into contact in a clean room ambient [114,115,116,117]. This results in spontaneous bonding between the activated materials at room temperature. In all three approaches, the cleaned surfaces are called the activated surfaces. The second and third approaches were developed for ionic materials to alleviate their surface activation induced polarization effect. In the fourth approach, the bonded specimens in the third approach are treated with an anodic bonding method (i.e., a voltage is applied on the bonded wafers during heating at low temperature) in air [118,119,120]. Although SAB has been demonstrated mainly for the bonding of hard materials, there are few results of SAB for flexible, soft, and hard materials. However, the fundamental understanding and innovative processes developed in SAB can be applied for the integration of these materials towards wearable sensing system applications. 

The primary challenges to the use of SAB to integrate flexible, soft, and hard materials are surface roughness incompatibility, high surface roughness materials requiring low external pressure/heat, materials deformation incompatibility, sensitivity of some material surfaces to plasma, and lack of theoretical understanding of bonding mechanisms. The approaches summarized here have shown significant promise in addressing these challenges, in particular the use of nanoadhesion layers and the use of reactive ion etching.

**Figure 5:**
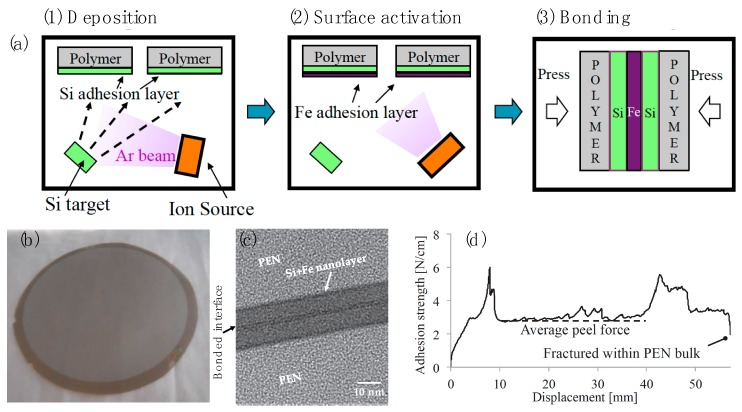
(**a**) Schematic diagram for surface-activated nanoadhesion layers-based bonding of thin polymers; (**b**) Optical image of the void-free interface of 100 mm in diameter PEN/PEN films; (**c**) High-resolution transmission electron microscope image of the bonded interface of PEN/PEN, showing a 20-nm-thick adhesion layer containing Si and Fe; and (**d**) Adhesion behavior of the PEN/PEN bonded interface as a function of displacement during the peel test. Bulk fracture in PEN near the bond interface was observed. From [112] (Copyright 2015 The Japan Society of Applied Physics).

Recent results have demonstrated bonding of flexible/flexible, flexible/hard, and soft/hard materials. The thicknesses of the flexible and hard materials used are in micrometers and, for the soft materials, are in millimeters. The second and third approaches offer better adhesion for the bonding of flexible materials and flexible/hard materials using nanoadhesion sites and hydroxyl molecules respectively. The bonding strengths of the flexible/hard materials in the second approach tend to be lower than that in the third bonding approach. The bonding strengths of the soft/hard materials in the third approach tend to be higher than that of the flexible/hard materials. The oxygen plasma increases reactive sites [121,122,123,124,125,126] on the activated surfaces. This provides improved surface energies, resulting in adhesion when contacted [121]. The adhesion can be further improved by heating the contacted surfaces at below glass transition temperatures [121]. In contrast, deposition of nanoadhesion layers causing chemical interactions between the activated surfaces can be used to increase the adhesion, as shown in Figure 5 [112]. In both cases, the influence of material type as well as plasma activation parameters on the bonding strength, is observed.

### 3.3. Summary

The integration of different materials to achieve the goal of reliable and robust wearable sensors is one of the key issues and has received considerable attention. A successful process must allow high resolution pattern definition and layer-to-layer alignment. It must be suitable to process heterogeneous materials with a wide range of chemical, mechanical, and thermal properties, and must achieve good adhesion in order for the overall structure to be robust to environmental exposure and mechanical deformation. In addition, it must do so with high throughput and scalability. The review by Gao and Cheng [127] specifically focuses on integration issues. 

## 4. Conclusions

We have given a concise overview of the issues pertinent to the incorporation and integration of heterogeneous materials needed for wearable sensors. Personal environment, health, and activity monitoring is the application area that has received the greatest attention in research on wearable sensors. Wearable sensors can be classified according to their configuration on the body, the level of integration of power and data processing, and the sensing function and method. In all cases, flexibility will be required and imperceptibility desired. Several innovative approaches have been explored to achieve these attributes. All the strategies available in the literature have in common the requirement to integrate heterogeneous materials—materials with a wide range of chemical, mechanical, and thermal properties. The materials involved include metals, conventional semiconductors, flexible polymers used as flexible substrates, in polymer electronics, or for the sensing function itself, and smart textiles. Methods proposed for integrating these materials include modifying conventional process steps to suit the properties of the materials in question, using transfer printing and multi-step processes to fabricate different parts of the system separately before integrating them into a final structure, using inkjet printing with inks that have been optimized to produce good resolution and adhesion and with alternative sintering processes for low temperature fabrication, and surface-activated bonding.

## Figures and Tables

**Table 1:** Summary of the range of materials needed in the design of wearable sensors together with some of the most important strategies for wearability, flexibility, and stretchability.

Material Category	Specific Examples of Materials	Application Areas	Challenges	Approaches to Flexibility and Stretchability
Metals	Ag/AgCl Cu/CuCl	Reference electrode (RE)/Ce in voltammetry	Deposition and patterning Reliability Fatigue due to bending and stretching	Using thin films on flexible substrates Placing the metal on the neutral mechanical plane (NMP) between flexible layers Folding out of plane
	Au	Contacts		
	Au/Cr Ti/Pt	Temperature measurements		
	Au	Working electrode (WE) for sensing Pb, Cu, and Hg		
	Bi	WE for sensing Pb, Cd, and Zn		
	Pd/PdO	WE for pH measurement		
	GaInSn	Strain		
Conventional Semiconductors and Dielectrics	Crystalline Si SOI	Electronics	Brittleness	Placing the semiconductors in the NMP Embedding small pieces in an open filamentary structure Using nanostructure materials (e.g., nanomembranes)
	Si nanomembranes	Strain		
	SiO_2_ Si_3_N_4_ HfO_2_	Dielectrics for transistors and capacitor structures		
Polymers	P3AT	Organic thin film transistors TFTs	Degradation due to environment Degradation due to exposure to analytes	Polymers are used because of their inherent flexibility Polyimide (PI) can be used in a multilayered structure to place brittle and hard materials in the NMP Capping materials can be used to protect the sensor from environmental and contaminant exposure
	PEDOT:PSS	Organic conductors		
	PDMS PI	Flexible substrates		
	PDMS	E-skin		
	PVDF	Flexible substrate, piezoelectric measurement, temperature measurement		
Textiles	Different fibers woven/knitted with polypyrrole/reduced graphene or ZnO	Strain measurement	Adhesion and structural integrity of the electronics	Coating nanofiber core with successive layers In situ polymerization Cooling, heating, and other treatments Pretreatment with layer to promote adhesion
2D materials	Graphene Functionalized graphene (rGO) rGO-nanomaterials composites (e.g., Pd–rGO and rGO/AgNWs)	Gas sensors	Same as conventional semiconductors	2D materials are ultrathin layers with excellent mechanical properties and hence better suited for flexible structures The use of the NMP and folding out of plane are still important strategies
